# Assessment of the forensic application of 50 Y-STR markers in a large pedigree

**DOI:** 10.1080/20961790.2020.1802827

**Published:** 2020-10-30

**Authors:** Yi Ye, Yuran An, Yiwen Yang, Hao Wu, Yuzi Zheng, Linchuan Liao

**Affiliations:** a West China School of Basic Medical Sciences & Forensic Medicine, Sichuan University, Chengdu, China; b Criminal Technology Department, Liupanshui Public Security Bureau, Guizhou, China

**Keywords:** Forensic sciences, forensic genetics, Y-STR, haplotype, rapid mutation, pedigree

## Abstract

Short tandem repeats on the Y chromosome (Y-STRs), characterized by paternal inheritance, are valuable in forensic practice. Notably, the potential application of Y-STRs in pedigrees should be drawn upon, especially in China’s surname-concentrated natural villages. The study focused on 50 Y-STRs, including 13 rapidly mutating (RM) Y-STRs that largely constitute the current Y-STR commercial kits, and determined the differences in these Y-STRs between branches in a large pedigree and the discriminatory power of these haplotypes in different units for male relatives. As indicated in the results, 14 inconsistencies were observed at 9 Y-STRs between 10 father-son pairs. In addition, these 50 Y-STR haplotypes discriminated 10 out of 47 father-son pairs, 106 of 148 cousin pairs, 70 of 119 uncle-nephew pairs, 17 of 39 brother pairs, and 14 out of 33 grandfather-grandson pairs in a large pedigree. The RM Y-STR set is able to differentiate close male relatives in a large pedigree.

## Introduction

With the genetic characteristics of paternal inheritance and lack of recombination, Y-chromosomal short tandem repeats (Y-STRs) are extremely useful tools in forensic practice [1]. In particular, they allow the unambiguous detection of male DNA components in mixtures with a high female background, as found in sexual assault or homicide cases. A falsely convicted male whose Y-STR profile does not match that of the sample from a crime scene is excluded [2, 3]. Other suspects or patrilineal relatives of the suspect can be identified by a Y-STR database search. Subsequently, when Y-STRs are combined with autosomal STRs, the perpetrator can be determined. This approach led to the apprehension of a serial killer in northwest China after 28 years [4]. In fact, during the review process of this manuscript, another 28-year-old Nanjing homicide case was solved based on the above technologies.

Plenty of China’s natural villages are surname-concentrated, in which thousands of residents with the same surname share paternal genetic ancestry and are grouped into numerous branches. A village has been reported where the residents have a single surname for dozens of generations. If a crime occurs in these places and the criminal happens to be a member of the patrilineal line, the power of conventionally used Y-STRs is insufficient to exclude numerous patrilineal relatives of the suspect. It would be time consuming to collect samples from thousands of male suspects. Recently, 13 rapidly mutating (RM) Y-STRs have been identified [5], and their ability to differentiate male lineages has been demonstrated [6, 7]. Some of them have been added to recently developed commercial kits, such as the Yfiler® Plus PCR Amplification Kit (Thermo Fisher Scientific, Waltham, MA, USA).

This study focused on 50 Y-STRs including RM STRs, found in the most common Y-STR commercial kits, to observe the variation in Y-STRs in a large pedigree, which will provide an advantage compared with simple mutation investigation.

## Materials and methods

### Samples and DNA extraction

**Figure 1.**
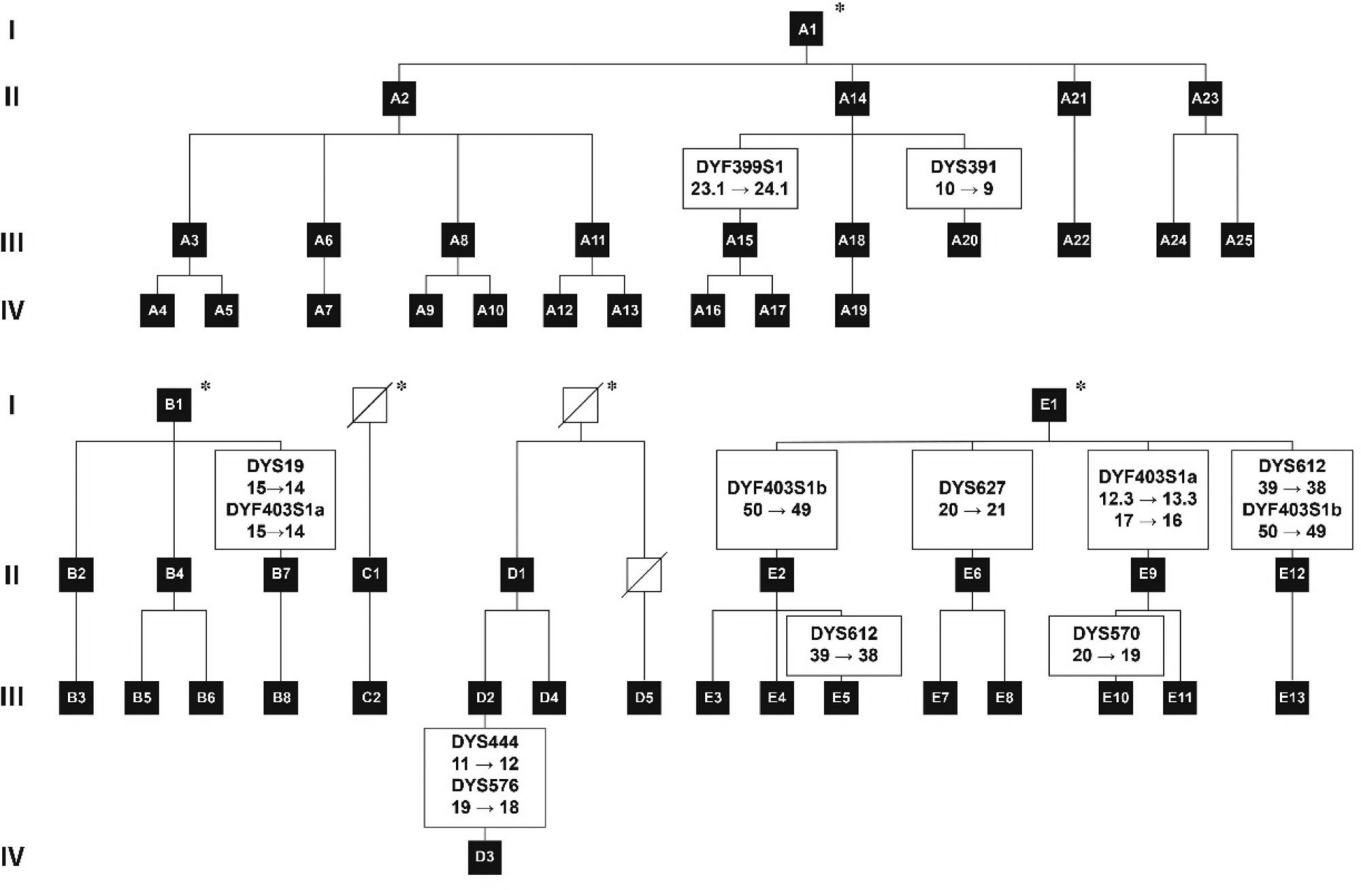
The pedigree chart. The black square represented the individuals involved in our study, while the white square plus a diagonal is opposite. The five individuals by an asterisk have the same father. Y-STRs with observed mutations are shown with the mutated alleles.

The samples were 53 male individuals from the four-generation pedigree, and they belonged to five branches designated A, B, C, D and E, as presented in Figure 1. Since the common ancestor had passed away long ago, three live individuals in the first generation were genotyped for 39 autosomal STRs (data not shown). The kinships between each pair were considered to be full siblings according to the biological full sibling identification code for practice in China [8].

Their bloodstains were collected on FTA Cards (Changchun Bokun Biotech CO., Ltd., Changchun, China). DNA was extracted by using Chelex 100 in a total volume of 100 μL without subsequent quantitation of the DNA amount [9]. Written informed consent was obtained from all study participants in accordance with the Humane and Ethical Research Principles of Sichuan University, and the study was approved by the Medical Ethics Committee of Sichuan University (Ethical approval number: K2019018).

### Y-STR genotyping

Thirty-seven conventional Y-STRs (DYS393, DYS446, DYS456, DYS522, DYS443, DYS520, DYS458, DYS481, DYS531, DYS19, DYS552, DYS391, DYS635, DYS437, DYS439, DYS389I/II, DYS388, DYS438, DYS447, DYS390, DYS510, DYS643, DYS587, DYS622, DYS533, Y_GATA_A10, Y_GATA_H4, DYS444, DYS385, DYS460, DYS630, DYS549, DYS392, DYS557, DYS448, DYS527, and DYS459) and 13 RM Y-STRs (DYF387S1, DYF399S1, DYF403S1a/b, DYF404S1, DYS449, DYS518, DYS526a/b, DYS547, DYS570, DYS576, DYS612, DYS626, and DYS627) were involved. Among them, one had “I” and “II” parts (i.e. DYS389) and two had “a” and “b” parts (i.e. DYS526 and DYF403S1). With the primer sequences attained from published literature, their exact map positions were retrieved adopting the UCSC in silico PCR tool (http://www.genome.ucsc.edu/cgi-bin/hgPcr).

These PCR units were amplified with four Y-STR multiplex PCR assays. Three of these multiplexes are commercially available, i.e. the AGCU Database Y24 STR Kit (Database Y24) [10], the AGCU Y SUPP Kit [11] (Y SUPP, AGCU ScienTech Incorporation, Wuxi, China) and the Yfiler® Plus Kit. The RM Y-STR set was designed according to Alghafri’s report [12]. PCR amplification was performed according to the manufacturer’s instructions or those provided by the corresponding reference.

PCR products were separated by multicapillary electrophoresis on the ABI Prism 3130 Genetic Analyzer (Applied Biosystems, Foster City, CA, USA). The fragment sizes were analysed using Gene-Mapper v.3.2. Allele designations were determined by comparing the sample fragments with those of allelic ladders from the kits or a ladder of RM Y-STRs made in our laboratory. The nomenclature adopted was that of the latest recommendations from the DNA Commission of the International Society of Forensic Genetics. RM Y-STR markers were named following the allele designation method adopted by Ballantyne [13].

### Quality control

For the Database Y24, Y SUPP, and RM set, 9948 was used as control DNA, while 007 was employed for Y-filer® Plus.

### Statistical analysis

The mutation events of 50 Y-STRs were observed using father-son pairs, and the mutant allele was evaluated for its potential application to distinguish among different branches in a pedigree. The 50 Y-STR markers were selected to constitute different units, including the minimum YHRD marker set, the various commercial kits (PowerPlex Y, PowerPlex Y23, Yfiler, Yfiler® Plus, Database Y24, Y SUPP), and the 13 RM Y-STR sets. For all 50 Y-STRs and each unit, the value was ascertained for differentiating the pairs of father-son, uncle-nephew, brothers, cousins, and grandfather-grandson.

## Results and discussion

The physical locations of the 50 Y-STR markers on the Y chromosome involved in this work are presented in Supplementary Figure 1. Seven of them are multi-copy systems (DYF403S1 with four copies and DYF399S1, DYF404S1, DYF387S1, DYS385, DYS527, and DYS459 with two copies each) and are associated with the palindromic sequences on the Y chromosome, especially in band q11.23. Two copies for DYS526 were observed, which did not comply with the number of physical locations along the Y chromosome per Y-STR predicted using the UCSC PCR tool. This was because the forward primer of DYS526 binds to two distinct areas, with a primer pair generating DYS526a and DYS526b as PCR products. In this regard, this phenomenon needs to be noted so that two or more peaks at this locus are not automatically interpreted as originating from a mixture of males. In addition, several STR loci, such as DYF403S1, are not simple sequence repeats [14], and the allele designation has not yet been unified. The diverse designation confines the alignment between different laboratories and hinders retrieval from the database. For these STR loci, acquiring sequence information using next generation sequencing technologies will be well studied in the future [15].

The genotypes of 50 Y-STRs from 53 individuals were successfully obtained. The overlapping Y-STR loci covered in different kits showed consistent genotypes, which indicated that these kits showed good compatibility. For the majority of Y-STRs, 41 genotypes remained consistent for all individuals from the four-generation pedigree. These Y-STRs could be considered characteristic surname markers. Fourteen inconsistencies were observed at the remaining nine Y-STRs and six of them were RM. All inconsistencies were one-repeat changes due to the occurrence of mutation events, as shown in Figure 1. The highest mutation rate was observed at the DYF403S1 locus (3.2 × 10^−2^), which has been found to be highly mutated in our previous mutation analysis on 100 father-son pairs [11].

There are a maximum of two Y-STR inconsistencies observed within the four father-son pairs. Our observation agrees with the recommended exclusion criteria of the minimum of three Y-STRs in paternity testing [16]. Naturally, the more Y-STR loci with high mutation rates that are investigated, the higher the likelihood to come across incompatible loci between the father and son pairs. In some circumstances, it is necessary to combine with other genetic markers to obtain a final decision of paternity exclusion [17].

Although autosomal STRs should be investigated first for differentiating close patrilineal relatives, Y-STRs, especially RM ones may be prioritized when analyzing male/female mixed samples. Provided that two close patrilineal relatives were involved in a suspected gang rape case, the Y-STR haplotype could be used to differentiate them. These 50 Y-STR haplotypes differentiated 10 out of 47 father-son pairs, marking a differentiation rate of 21.3%; moreover, they could differentiate 106 out of 148 cousin pairs, 70 out of 119 uncle-nephew pairs, 17 out of 39 brother pairs, and 14 out of 33 grandfather-grandson pairs. The differentiation rate rises in line with the degree of kinship. The distant relatives indicate considerable meiosis and a higher probability of mutation events, which can occur at each meiosis independently. In summary, the 50 Y-STR haplotype allowed 56.2% of these relative pairs to be separated. For other units that are part of the Y-STRs among 50 units, the number of pairs with inconsistencies and differentiation rates between two related men for different units are listed in Table 1. Thirteen RM Y-STR haplotypes played a dominant role, which accounted for 93.5% of the differentiation rates. Minimal, PowerPlex Y and Yfiler had the poorest performance, only allowing the separation of 10.6% of these relative pairs. The feasibility of the RM Y-STR haplotype for differentiating male relatives was sufficient, as indicated by the higher number of mutations within the pedigrees than in other units.

**Table 1.** The number of pairs with the inconsistency and differentiation rates between two related men for different units.

	Minimal	PowerPlex Y	PowerPlex Y23	Yfiler	Yfiler^®^ Plus	DATA Y24	Y SUPP	RM	All
Father-son (47 pairs)	2 (4.3)	2 (4.3)	4 (8.5)	2 (4.3)	6 (12.8)	3 (6.4)	1 (2.1)	9 (19.1)	10 (21.3)
Uncle-nephew (119 pairs)	13 (10.9)	13 (10.9)	18 (15.1)	13 (10.9)	53 (44.5)	14 (11.8)	19 (16.0)	66 (55.5)	70 (58.8)
Brothers (39 pairs)	4 (10.3)	4 (10.3)	5 (12.8)	4 (10.3)	13 (33.3)	4 (10.3)	2 (5.1)	16 (41.0)	17 (43.6)
Cousins (148 pairs)	20 (13.5)	20 (13.5)	28 (18.9)	20 (13.5)	86 (58.1)	20 (13.5)	42 (28.4)	99 (66.9)	106 (71.6)
Grandpa-grandson (33 pairs)	2 (6.1)	2 (6.1)	4 (12.1)	2 (6.1)	7 (21.2)	3 (9.1)	1 (3.0)	13 (39.4)	14 (42.4)
Sum (386 pairs)	41 (10.6)	41 (10.6)	59 (15.3)	41 (10.6)	165 (42.7)	44 (11.4)	65 (16.8)	203 (52.6)	217 (56.2)

In conclusion, for male/female mixed samples, the question remains of whether the sample is from the perpetrator or whether different but related men were involved. The subsequent analysis of the RM Y-STRs will provide further evidence for the exclusion of close male relatives.
